# Society Meetings

**Published:** 1893-10

**Authors:** 


### Society Meetings.

THE Mississippi Valley Medical Association will hold its nineteenth annual meeting at Indianapolis, Wednesday, Thursday, and Friday, October 4, 5, and 6, 1893. President, R. Stansbury Sutton, M. D., Pittsburg; Vice-Presidents, W. N. Wishard, M. D., Indianapolis; W. S. Christopher, M. D., Chicago; Secretary, Frederick C. Woodburn, M. D., Indianapolis; Committee of Arrangements—George J. Cook, M. D., Chairman; H. M. Lash, M. D.; O. G. Pfaff, M. D.; Theo. Potter, M. D.; George W. Vernon, M. D.

The following titles of addresses and papers have been offered, namely: President's address, Fibroid Tumors of the Uterus, R. Stansbury Sutton, M. D., Pittsburg; address on Medicine, James F. Hibberd, M. D., Richmond, Ind.; address on Surgery, The Anatomy and Surgical Treatment of Inguinal Hernia in the Male, Henry O. Marcy, M. D., Boston.

Papers will be read in the different sections as follows: S. E. Allen, Cincinnati, The Pathology of Pharyngeal Inflammations; E. Wyllis Andrews, Chicago, A Surgeon's View of Appendicitis; John Aulde, Philadelphia, Cellular Therapy, Its Practical Adaptation in the Treatment of Disease; Robert H. Babcock, Chicago, The Schott Method of Treating Diseases of the Heart by Means of Baths and Gymnastics; A. J. Banker, Columbus, Ind., Some Practical Points in the Treatment of Abscesses and Tuberculous Glands; A. D. Barr, Calamine, Ark., The Physiology of Conception; Adolph Blitz, Indianapolis, Pterygium, Its Nature and Treatment; Joseph L. Bauer, St. Louis, The Treatment of Hip-Joint Disease as Related to its Etiology; Louis Bauer, St. Louis, Incurability of Advanced and Extreme Cases of Talipes Equino-Varus by the Means and Methods in Vogue at the Present Time—Suggestion of a Way to Remove the Deformity Without Disturbing the Usefulness of the Extremity; J. T. Berghoff, St. Joseph, Mo., Treatment of Fractures of the Leg; Seth S. Bishop, Chicago, Treatment of Mastoid Diseases, with Operation ; I. N. Bloom, Louisville, Electrolysis in the Removal of Superfluous Hairs ; A. W. Brayton, Indianapolis, Presentation of Cases of Skin Disease, (*a*) Sarcoma of the Face, with Sections, (*b*) Lupus Mutilans, (*c*) Xeroderma Pigmentosum, two cases ; J. G. Carpenter, Stanford, Ky., Strictures of Large Caliber, Follicular Urethritis, Urinary Infiltration, Abscess —two cases—recovery ; A. Morgan Cartledge, Louisville, Shall We Operate in All Cases of Appendicitis ? Wm. Cheatham, Louisville, Medical Ophthalmoscopy ; Joshua Chitwood, Connersville, Ind., The Old and New Method as Applied to Surgery ; C. G. Comegys, Cincinnati, Medical Jurisprudence ; Wm. T. Corlett, Cleveland, Pemphigus, Its Varieties, Course, and Treatment, with a Report of Some Unusual Cases ; T. D. Crothers, Hartford, Conn., The Medical Treatment of Inebriety ; N. D. Cox, Spencer, Ind., Puerpera Hemorrhagica, with Report of a Case ; J. C. Culbertson, Cincinnati, Diphtheria ; Ephraim Cutter, New York, The Treatment of Sclerosis of the Spine ; Wm. H. Davis, Denver, Col., Paper ; Wm. B. Dewees, Salina, Kan., The Erect Posture for Gynecological Examinations ; Allen De Vilbiss, Toledo, O., New Devices for Cutting Bone ; Arch. Dixon, Henderson, Ky., Paper ; L. H. Dunning, Indianapolis, Intestinal Obstruction After Abdominal Section ; Joseph Eichberg, Cincinnati, Essential Paroxysmal Tachycardia ; Orpheus Everts, College Hill, O., Problems of Public Interest Concerning the Insane ; Wm. A. Galloway, Xenia, O., Diphtheria, A Treatment Giving a Low Death-rate in Hospital and Private Practice ; Heneage Gibbes, Ann Arbor, Mich., The History of a Case of Phthisis Treated with Gold and Iodine, and where Inoculations of Guinea Pigs with the Sputum were kept up until it became Inocuous ; Rufus B. Hall, Cincinnati, Paper ; D. J. Hayes, Milwaukee, Some Points on Surgery of the Prostate ; F. C. Heath, Indianapolis, Hygiene of the Eye ; Fred Jenner Hodges, Anderson, Ind., Continuous Submersion in Infected Wounds of the Extremities ; T. E. Holland, Hot Springs, Ark., Paper ; Bayard Holmes, Chicago, What Sort of a Medical Education is Required, and Whose Duty is it to Furnish it ? Wm. H. Humiston, Cleveland, The Treatment of the Diseases of the Uterine Appendages ; Wm. F. Hutchinson, Providence, R. I., Electric Anesthesia, Further Studies ; Geo. F. Keiper, Lafayette, Ind., Etiology of Deafness, and Its Prevention ; J. H. Kellogg, Battle Creek, Mich., A Critical Study of the Symptomatology of the Disorders of Digestion ; G. W. H. Kemper, Muncie, Ind., A Case of Senile Gangrene, Treated by Amputation ; Emory Lanphear, Kansas City, Surgery of the Cranium, What is the Proper Treatment? Hugh M. Lash, Indianapolis, Chorea, Its Etiology and Treatment ; Bransford Lewis, St. Louis, The Pathological Anatomy of Urinary Retention, with Deductions as to Treatment ; Wm. H. Link, Petersburg, Ind., The Value of a Close Observation of Other Men's Work ; H. W. Loeb, St. Louis, Some Illustrative Cases of Nasal Headache ; I. N. Love, St. Louis, Mo., Chorea in its Relation to Rheumatism ; G. Frank Lydston, Chicago, Some Heresies Regarding Prostatic Pathology ; Anne H. McFarland, Jacksonville, Ill., The Classification of the Insane ; Chas. F. McGahan, Bethlehem, N. H., Physical Culture in Pulmonary Disease ; Theo. A. McGraw, Detroit, Paper ; L. S. McMurtry, Louisville, Paper ; Joseph M. Mathews, Louisville, Ulceration of the Rectum, Its Etiology and Treatment ; J. G. Meachem, Racine, Wisconsin, Lung Diseases as they Occur on the Shores of Lake Michigan ; Giles S. Mitchell, Cincinnati, Ohio, Cesarian Section and its Substitutes ; J. McLean Moulder, Kokomo, Ind., Brain Surgery, with Report of Cases ; J. B. Murphy, Chicago, Perforative Peritonitis ; Frank P. Norbury, Jacksonville, Ill., Medico-Legal Aspect of Brain Tumors ; A. H. Ohmann-Dumesnil, St. Louis, Chancroid of the Eyelid ; J. C. Oliver, Cincinnati, Tubercular Disease of the Tarsus, Surgical Treatment, Results ; L. F. Page, Indianapolis, Hay Fever ; H. O. Pantzer, Indianapolis, Tubercular Peritonitis ; Theo. Potter, Indianapolis, The Pathology and Principles of Treatment of Asthma ; Joseph Price, Philadelphia, Why Gynecology and Obstetrics Should be in the Hands of Specialists ; I. N. Quinby, Jersey City, N. J., A New Method of Operating at the Ankle-Joint for Injuries of the Foot ; A. Ravogli, Cincinnati, Cutaneus Psorospermosis ; J. M. Ray, Louisville, The Nose and Naso-Pharynx in Their Relation to Suppurative Diseases of the Middle Ear ; Thad. A. Reamy, Cincinnati, The Evolution in the Treatment of Uterine Fibroids Since my Entrance into the Profession ; B. Merrill Ricketts, Cincinnati, Report of Surgical Cases, with Photographs ; John Ridlon, Chicago, Differential Diagnosis and Principles of Treatment of Hip-Joint Disease ; Eric E. Sattler, Cincinnati, Paper ; J. C. Sexton, Rushville, Ind., Study of a Fatal Case of Essential Tachycardia ; M. T. Scott, Lexington, Ky., Septic Infection of the New-Born ; Y. C. Smythe, Greencastle, Ind., Dirt vs. Drugs in the

Treatment of Diabetes Mellitus; Albert E. Stearne, Indianapolis, Multiple Sclerosis; F. E. Stewart, Watkins, N. Y., Some of the Treatments Employed in Sanitariums; C. B. Stemen, Fort Wayne, Ind., Antiseptic Precautions in Railway Injuries; Leon Straus, St. Louis, A Plea for More Frequent and Earlier Colotomy in Painful Malignant Diseases of the Rectum; Frank J. Thornbury, Buffalo, N. Y., The Bacteria of the Surface—Disinfection of the Latter—Non-Utility of Antiseptics; Max Thorner, Cincinnati, Modern Methods of Treating Ear Diseases; Lyman Beecher Todd, Lexington, Ky., Certain Diseases of Infancy, Their Prevention; Geo. W. Vernon, Indianapolis, Infantile Therapeutics; Carl H. Von Klein, Cleveland, Nasal and Post-Nasal Vegetations; Karl Von Ruck, Asheville, N. C., Paper; Edwin Walker, Evansville, Ind., Reflex Irritation as a Cause of Disease; H. O. Walker, Detroit, Kraske's Operation, with Report of Cases; Geo. W. Webster, Chicago, The Value of an Examination of the Blood as an Aid in Diagnosis; X. O. Werder, Pittsburg, The Present Status of the Treatment of Uterine Fibroids; J. R. Weist, Richmond, Ind., The Diagnosis and Treatment, by the General Practitioner, of the Minor Diseases of the Sexual Organs of Women; Wm. E. Wirt, Cleveland, Treatment of Old and Neglected Cases of Hip Disease; Wm. N. Wishard, Indianapolis, Paper; E. A. Wood, Pittsburgh, Therapy of Gold; J. E. Woodbridge, Youngstown, O., Can Typhoid Fever be Aborted?

---

AT THE meeting of the Ohio State Medical Society, held in June, the following officers were elected: President, N. P. Dandridge, M. D., Cincinnati; First Vice-President, F. C. Larimore, M. D., Mt. Vernon; Second Vice-President, Wm. Caldwell, M. D., Fremont; Third Vice-President, W. T. Corlett, M. D., Cleveland; Fourth Vice-President, L. S. McCurdy, M. D., Dennison; Secretary, Thos. Hubbard, M. D., Toledo; Assistant Secretary, Charles Graefe, M. D., Sandusky; Treasurer, J. A. Duncan, M. D., Toledo.

---

THE MEDICAL SOCIETY OF THE STATE OF NEW YORK.—The following Business Committee has been appointed by the President of this Society, Dr. Bendell,—namely: Dr. Henry Flood, of Elmira; Dr. L. Bolton Bangs, of New York; Dr. Edward Clark, of Buffalo—to whom communications may be addressed regarding papers for the next meeting of the society, in February next.

F. C. CURTIS, *Secretary.*

